# Application of serum exosomal hypoxia-inducible factor-1 alpha (HIF-1α) as potential circulating biomarker for bacterial peritonitis

**DOI:** 10.1080/21655979.2021.2006866

**Published:** 2022-01-14

**Authors:** Xiao-Yi Chen, Qian-Long Wang, Wei-Xue Huang, Long-Wei Li, Lan-Cong Liu, Yi Yang, You-Jiao Wu, Shan-Shan Song, Hao Ma, Hua Zhou, Pei Luo

**Affiliations:** a State Key Laboratories for Quality Research in Chinese Medicines, Macau University of Science and Technology, Macau, China; b School of Materials Science and Engineering, Tsinghua University, Shaw Technical Science Building, Beijing, China; c State Key Laboratory of Bioorganic and Natural Products Chemistry, Center for Excellence in Molecular Synthesis, Shanghai Institute of Organic Chemistry, Chinese Academy of Sciences, Shanghai, China

**Keywords:** Bacterial peritonitis, hypoxia, HIF-1α, biomarker, exosome

## Abstract

Bacterial peritonitis is a severe disease that diagnosis remains challenging for clinicians. Measuring biomarkers might be a rapid diagnostic method. The objective of this study was to analyze and evaluate the dynamic changes in HIF-1α concentration in serum exosomes during bacterial peritonitis. The pre-clinical application value of serum exosomal HIF-1α was evaluated via imipenem and cilastatin sodium (ICS) intervention in the bacterial peritonitis model. The new colorimetric method to quantitate dynamic expression changes of HIF-1α in serum exosomes during bacterial peritonitis was established by our team via using the gold seed-coated with aptamer-functionalized Au @ Au core-shell peroxidase mimic. The typical inflammatory cytokines of bacterial peritonitis were also measured. Following intramuscular administration with ICS, *In-Vivo* Xtreme imaging system was used to visualize abdominal infection extent. Meanwhile, HIF-1α concentration in rat serum exosomes and pro-inflammatory factors levels in serum were detected. The serum typical inflammatory cytokines levels were elevated in GFP-labeled *E.coli* induced bacterial peritonitis. The serum exosomal HIF-1α levels clearly increased at 12 h, reached the peak during 24–48 h, and then gradually decreased at 72 h. Following intramuscular administration with ICS, the abdominal infection extent, HIF-1α concentration in serum exosomes, and the serum pro-inflammatory factors levels were reduced at 24 h in GFP-labeled *E. coli* induced bacterial peritonitis model. The serum exosomal HIF-1α can be used as a biomarker in the early stage of bacterial peritonitis, which might provide the basic research in the pre-clinical for further predicting and monitoring the pathological process of bacterial peritonitis.

## 1. Introduction

Bacterial peritonitis is an inflammation of the peritoneum by microorganisms such as Gram-negative bacilli. It is usually developed in decompensated cirrhosis patients or patients receiving continuous ambulatory peritoneal dialysis therapy. It is the host’s systemic inflammatory response to bacteria [1]. Complicated Intra-Abdominal Infection Observational Worldwide (CIAOW) shows that the total mortality of patients with peritonitis worldwide is 10.5% [2]. The most widely used in clinical practice for routine detection of bacterial peritonitis is diagnostic abdominal paracentesis, including neutrophil count and ascites culture examination. However, the method of diagnostic abdominal paracentesis is time-consuming (24–48 h) and may be involved in severe abdominal wall hematomas, such as abdominal bleeding or intestinal needle puncture [3]. Therefore, improving the accuracy approaches for the early prediction of bacterial peritonitis and the research of early diagnostic biomarkers by applying noninvasive liquid biopsy are still warranted. The exosomes is a vital part of the extracellular micro-environment, which shows significant superiority over other liquid biopsy sources [4,5]. Since discovering that exosomes contain various active biomolecules (protein and nucleic acid transformation), interest has gradually increased [6]. Exosomes can be released into the extracellular environment through paracrine and other ways, which play an essential role in signal transduction and material transfer between cells in various ways [7]. Since exosomes released from different tissues can be collected from body fluids, their cargo represents possible disease markers and the means of treatment [8]. Evidence shows that the level of exosomes containing proteins or miRNAs in the systemic circulation may reflect tissue damage related to tissue hypoxia caused by bacterial infection [9]. Exosome contents have excellent potential for fluid biopsy development. They may replace many invasive tissue biopsies, particularly as an early biomarker for bacterial peritonitis [10].

Hypoxia-inducible factor 1-alpha (HIF-1α), the primary regulator of cell response to hypoxia [11], has been reported to be widely involved in the mechanism of hypoxic metabolism and has become an essential regulator of bacterial infection [12]. When immune cells are recruited into inflammatory tissue, it encounters a decreasing oxygen gradient, which increases the expression of HIF-1α in the cell and initiates the activation of genes with pro-inflammatory and bactericidal effects [13]. HIF-1α is induced by bacterial infection and promotes the increase of phagocytosis and the release of antimicrobial peptides and granule proteases with direct microbicidal activity [14]. Myeloid cells deficient in HIF-1α mice are more susceptible to infection, fail to restrict the systemic spread of infection [15], and reduce phagocytes’ killing ability against Gram-negative and Gram-positive bacteria [16]. Epidemiological investigations have found that sepsis is sometimes caused by bacterial peritonitis [17]. Mingmin Fu et al. [18] used bioinformatic analysis to find that the gene of HIF-1α were found significantly upregulated in patients with sepsis. These studies indicated that HIF-1α had different vital roles at various times during bacterial peritonitis [19]. The association between the induction of HIF-1α expression and exosome release has been noted, but the direct relationship between quantitative dynamics changes of HIF-1α and the severity of bacterial peritonitis is still unclear. This is due to the instability of HIF-1α in body fluids, which makes the detection of HIF-1α in bacterial peritonitis infeasible. The relative stability of HIF-1α carried in exosomes due to the lack of degradative enzymes allows for direct quantification or dynamic monitoring even during exosome release in bacterial peritonitis. Although the commercial ELISA kits, colorimetric assays, and western bolt can enable the determination of HIF-1α in tissues or plasma, their detection limitation cannot meet the actual needs for detection in exosomes containing HIF-1α. Our team established a new colorimetric method of HIF-1α in serum exosomes using the gold seed-coated with aptamer-functionalized Au @ Au core-shell peroxidase mimic [20]. The detection limit of this method is 0.2 ng/L which significantly improves the detection sensitivity to HIF-1α in serum exosomes [20]. ICS is the combination of imipenem and cilastatin used in clinical to treat various serious infections, including bacterial infections of the abdominal cavity and urinary tract and sepsis [21,22]. Imipenem is a carbapenem beta-lactam broad-spectrum antibiotic that could protect against various gram-negative and positive bacteria [23]. The present study aims to analyze and evaluate the dynamic changes in the quantitative expression and distribution of HIF-1α in serum exosomes during bacterial peritonitis through the gold seed-coated with aptamer-functionalized Au @ Au core-shell peroxidase mimic. Meanwhile, whether ICS intervention can ameliorate bacterial peritonitis infection through antibacterial effects and reducing the expression of HIF-1α in serum exosomes to further evaluate the possible value of HIF-1α as the potential biomarker in the pre-clinical application of the early stage of bacterial peritonitis, it might provide a research basis for the early diagnosis of the process of acute bacterial peritonitis.

## 2. Methods

### 2.1 Chemical and reagent

Kanamycin, sodium chloride (NaCl), 3,3ʹ5,5ʹ-tetramethyl benzidine (TMB), sodium dodecyl sulfate (SDS), and dichlorodimethylsilane were purchased from Aladdin Bio-Chem Technology Co., Ltd (Shanghai, China); Rat IL-6, IL-1β, and TNF-α ELISA kit were purchased from Multi science (lianke) biotech, Co., Ltd (Hangzhou, China); HRP-labeled secondary antibodies were purchased from Santa Cruz Biotechnology, Co., Ltd (USA); HIF-1α polyclonal antibody and HIF-1α C domain (number of amino acids 297, molecular weight 32.7 KDa) was synthesized in house; Anti-CD63 antibody was purchased from Abcam Trading Co., Ltd (Shanghai, China); Bovine serum albumin (BSA), Tris (2-carboxyethyl) phosphine (TCEP), N,N,N’,N’-tetramethyl ethylenediamine (TEMED), and ammonium persulfate (APS) were purchased from Sigma-Aldrich Co., Ltd (USA); Imipenem and Cilastatin Sodium were purchased from Meilun Biotechnology Co., Ltd (Dalian, China); 30% Acrylamide/bis solution 29:1 (3.3% crosslinker) solution and precision plus protein standards dual color were purchased from Bio-Rad Laboratories (China); Phosphate Buffered Saline tablets and RIPA lysis buffer were purchased from Thermo Fisher Scientific Inc (USA); Sulfhydryl modified hypoxia inducible factor-1α (HIF-1α) aptamer 5ʹ-HS-(CH2)6-CCCACCCACCCATGTTGTTGTCTACGTGCT-3ʹ was purchased from Sangon Biotech Co., Ltd (Shanghai, China). All the chemicals used in this research are analytical reagent.

### 2.2 Animals

Male Sprague-Dawley rats (180–220 g) and female BALB/c mice (20–30 g) were purchased from the Laboratory Animal Services Center, the Chinese University of Hong Kong. According to the standard diet for animals. The animals were acclimated in a moderate and light (12 h light/dark cycle) environment of the specific pathogen-free (SPF) laboratory with a suitable room temperature (22 °C ± 1 °C), and the relative humidity was set as 40 ~ 70%. All animals had free access to standard food and water. The local Animal Ethics Committees approved animal experiments of the State Key Laboratories for Quality Research in Chinese Medicines, Macau University of Science and Technology.

### 2.3 Model of bacterial peritonitis and serum sample collection

*Escherichia coli* BL21 (DE3) labeled green fluorescent protein (GFP) (Novagen, Germany) was cultured in Luria–Bertani (LB) medium at 37°C for 200 rpm to resuscitate the GFP-labeled *E.coli*. GFP-labeled *E.coli* was harvested at the mid-log phase and washed twice with sterile saline before injection to clear the LB medium. Rats were randomly divided into two groups, and each group comprised of 10 animals which are control and model groups. The model group was injected by intraperitoneal challenge with GFP-labeled *E.coli* (5 × 10^8^ colony-forming units [CFU]) in 300 μL sterile saline to induce the acute bacterial peritonitis. At the time point of 0, 6, 12, 24, 48, and 72 h after the injection of GFP-labeled *E.coli*, blood was continuously taken from the orbit. The blood was allowed to clot at room temperature for 1 h and then centrifuged at 3,000 × g for 10 min for serum preparation. Serum samples are stored at −20°C until the subsequent use. After the last blood collection, the rats in the control and the model groups were sacrificed by intraperitoneal injection of sodium pentobarbital (2%, 3 mL/kg).

### 2.4 Drug treatment

Thirty male rats were randomized into three groups and each group comprised of 10 animals that were control, model, and ICS (20 mg/kg) group. GFP-labeled *E.coli* (3 × 10^8^ CFU, 300 μL) was injected intraperitoneal except those of control groups. The solution of sterile saline was given to the control group. Two hours after intraperitoneal injection of GFP-labeled *E.coli*, ICS (20 mg/kg) was intramuscular injection in the ICS group. At 0 and 24 h after the injection of GFP-labeled *E.coli*, blood was continuously taken from the orbit. The blood was allowed to clot at room temperature for 1 h, and then centrifuged at 3,000 × g for 10 min for serum preparation. Serum samples are stored at −20°C until the next testing. After the last blood collection, the rats in the control and the model groups were intraperitoneally injected with sodium pentobarbital (2%, 3 mL/kg) to sacrifice the rat.

### 2.5 Cytokine assay

The levels of interleukin 6 (IL-6), interleukin 1beta (IL-1β), tumor necrosis factor-alpha (TNF-α) in the serum were measured enzyme-linked immunoassay (ELISA) kits according to manufacturer’s instructions.

### 2.6 Isolation and purification of exosomes

The method of extraction and purification of exosomes from serum by ultracentrifugation is described with reference to previous literature and with a slight modification [24–26]. Briefly, the blood was allowed to be put at room temperature for 1 h, the blood was centrifuged at 2,000 × g for 10 min at room temperature to extract the serum immediately. Next, the samples were centrifuged again to remove cells and other debris in the serum at 3,000 × g for 10 min at room temperature. Subsequently, serum was diluted with PBS (1:1) and centrifuged at 10,000 × g for 30 min at 4°C. Then the supernatant was filtered through the 0.22 *µ*m filter (Millipore, Billerica, MA) to remove the micro-vesicles that were larger than exosomes. Then the supernatant was ultracentrifuged again at 100,000 × g for 90 min at 4°C by using Sorvall WX100 Ultracentrifuge (Thermo Fisher Scientific, USA). Finally, the supernatants were gently decanted, and the exosomes sediments were washed once in PBS and ultracentrifuged again at 100,000 × g for 90 min at 4°C. The exosomes pellets were dispersed in 200 μL of PBS for sample determination and stored at −80°C until further experiments.

### 2.7 Transmission Electron Microscopy (TEM)

Transmission electron microscopy (TEM) was used to examine the purified exosomes as described previously [27]. Purified exosomes were resuspended in 100 μL phosphate-buffered saline (pH 7.4) containing 1% glutaraldehyde and then loaded onto formvar/carbon-coated electron microscopy copper grids dry at room temperature for 10 min. Then it was stained with 2% phosphotungstic acid solution for 2 min at room temperature. Subsequently, the grid was further dried at room temperature for 10 min to remove the excess liquid. FEI Tecnai G20 transmission electron microscope (FEI Co., Holland) was used to visualize the images at an accelerating voltage of 120 kV.

### 2.8 Nanoparticle Tracking Analysis (NTA)

Nano Sight NS300 and Malvern Nano Sight NTA 3.0 software (Malvern, UK) were used to analyze the concentration and size distribution of the isolated exosomes according to the previously established method [28]. Briefly, the purified exosomes solution was vortexed followed by serial dilution of sterile Phosphate saline buffer at concentrations ranging from 2 × 10^8^ to 2 × 10^9^ particles/mL. The exosomes sample solution was introduced into the sample chamber by Nano Sight NS300. The movement of pellets under Brownian motion was recorded for 1 min to analyze the parameters of particle concentrations and size distribution profiles.

### 2.9 Western blotting for analysis

CD63, a specific exosome surface marker, was detected by Western blot [29]. RIPA buffer was used to lysis the exosomes pellets to extract the total exosomes proteins. Bicinchoninic acid (BCA) kit was used to identify the protein concentration. Exosome’s proteins (10 μg) were run and separated by 10% SDS-PAGE. Then, it was transferred to the PVDF membrane. TBST containing 5% BSA was used to block the membranes for 1 h and room temperature. Afterward, the primary antibody anti-CD63 (1:1000) was used to incubate at 4°C overnight. The membrane was washed three times and incubation with an appropriate secondary antibody for 1 h at 37°C. The protein bands were visualized by Imaging systems Amersham Imager 600 scanner (General Electric Company, USA) and analyzed with ImageJ software (version 1.8.0, National Institutes of Health).

### 2.10 Determination of HIF-1α in serum exosomes

The colorimetric detection method of HIF-1α in serum and serum exosomes using the gold seed-coated with aptamer-functionalized Au @ Au core-shell peroxidase mimic as described previously, established by our team [20]. Briefly, 100 μL of the HIF-1α polyclonal antibody solution (0.01 mg/ml) was added to each well of 96-well plates at 4°C for 18 h in order to prepare the HIF-1α polyclonal antibody-coated ELISA plates. Then 1% bovine serum albumin solution (200 μL) was added to each well of 96-well plates at 37°C for 2 h and washed three times with PBS, vacuum dried, and placed at 4°C for later use. Subsequently, the sample (50 μL) was added to 96-well plates and incubated at room temperature for 30 min. Then AuNPs-aptamer solution (50 μL) was added to 96-well plates and continue to set for 30 min. After washing it three times with PBS, the gold seed growth solution (100 μL) was added to each well and incubated at 28°C for 20 min. Next, the plates were washed three times with PBS and dried. At last, the substrate solution (100 μL) was mixed with TMB solution (500 μL) and H_2_O_2_ (320 μL)) and then added the mixture to each well and continue to incubate for 15 min. SpectraMax paradigm multi-mode microplate reader (Molecular Devices, Sunnyvale, CA, USA) was used to measure the absorbance of each well at 652 nm (OD_652nm_).

### 2.11 Whole-body imaging of GFP-labeled E.coli in bacterial peritonitis

The outside noninvasive imaging technique to visualize the infection process in infected animals with GFP-expressing bacteria is described with reference to previous literature and with a slight modification [30,31]. *In-Vivo Xtreme imaging system (*Bruker, Billerica, MA) was used to conduct the bioluminescent imaging with a CCD camera with a filter of 700 nm. Briefly, thirty female BALB/c mice were randomly divided as aforementioned. GFP-labeled *E.coli* (3 × 10^8^ CFU, 300 μL) was injected intraperitoneal except for control groups to induce acute bacterial peritonitis model. The solution of sterile saline was given to the control group. Two hours after intraperitoneal injection of GFP-labeled *E.coli*, ICS (20 mg/kg) were intramuscular injections in the ICS group. All experiment groups were imaged with *In-Vivo Xtreme imaging system* to observe the mice whole-body fluorescence imaging of GFP-labeled *E.coli* after the 6, 12, and 24 h post-injection. Mice were anesthetized with isoflurane using a vaporizer, and the mice fluorescent image was obtained by GFP filter set (excitation wavelength: 475 nm; emission wavelength: 515 nm).

### 2.12 Statistical analysis

All experimental data are expressed as mean ± S.E.M, and each experiment is repeated at least three times. Graph Pad Prism 5.0 software (Graph Pad Software Inc., La Jolla, CA, United States) was used for one-way analysis of variance to analyze the statistical significance between the groups. P < 0.05 was considered statistically significant.

## 3. Results

This research preformed GFP-labeled *E.coli* induced bacterial peritonitis rat model to detect the dynamic changes in HIF-1α concentration in serum exosomes during bacterial peritonitis via using the gold seed-coated with aptamer-functionalized Au @ Au core-shell peroxidase mimic for the first time. Then, the typical inflammatory cytokines of bacterial peritonitis were also measured. The quantification of serum exosomes allows the early detection and follow-up of bacterial peritonitis during treatment, as demonstrated in a rat model of bacterial peritonitis treated with ICS. Following intramuscular administration with ICS, the abdominal infection extent, HIF-1α concentration in serum exosomes, and the serum pro-inflammatory factors levels were reduced at 24 h in GFP-labeled *E. coli* induced bacterial peritonitis model. Therefore, the serum exosomal HIF-1α may be used as a biomarker in bacterial peritonitis.

### 3.1 Typical inflammatory cytokines of bacterial peritonitis rat models

**Figure 1.**
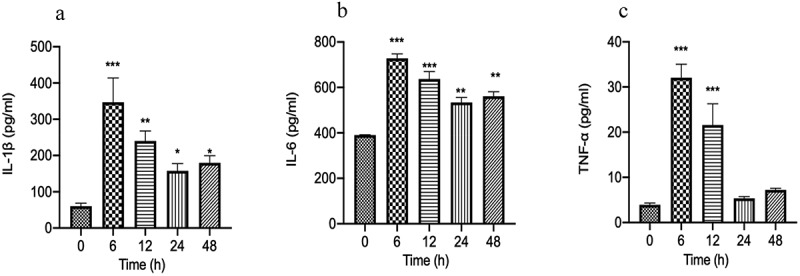
The typical inflammatory cytokines (IL-6, IL-1β, TNF-α) in serum of GFP-labeled *E.coli* induced bacterial peritonitis rat models were detected by ELISA kits at 0, 6, 12, 24, and 48 h (n = 10 rats per group). a: The concentration of IL-1β in serum; b: The concentration of IL-6 in serum; c: The concentration of TNF-α in serum. Experimental values are expressed as mean ± S.E.M. *P < 0.05, **P < 0.01, and ***P < 0.001 compared with time 0.

GFP-labeled *E.coli* is often used to induce bacterial peritonitis models [32]. Immune system disorder is one of the main consequences of bacterial infection. To the expression of inflammatory factors in GFP-labeled *E.coli* induced bacterial peritonitis episodes, the commercially available ELISA kits were used to detect the level of pro-inflammatory factors in the serum at the time point of 0, 6, 12, 24, and 48 h. As shown in Figure 1, levels of TNF-α, IL-1β, and IL-6 were increased to the peak level at 6 h when compared with baseline levels at 0 h of the episode (P < 0.001). The levels of pro-inflammatory cytokines (IL-1β, IL-6) are remained higher than the baseline levels at 0 h of the episode at 48 h (Figure 1a and b). A strongly declined was detected in the serum level of TNF-α in GFP-labeled *E.coli*-induced bacterial peritonitis rats. In particular, TNF-α level declined rapidly to reach baseline levels at 24 h the episode (Figure 1c). The transient increases in the IL-6, TNF-α, and IL-1β levels storm prior, consistent with the excessive inflammation in bacterial peritonitis.

### 3.2 Identification of the isolated serum exosomes

**Figure 2.**
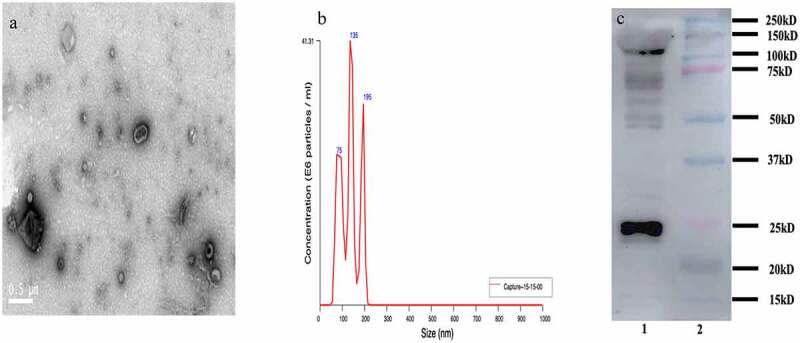
Characterization of exosomes isolated from the serum in bacterial peritonitis rat models; a: Transmission electron microscopy (TEM) characterization of the morphology exosomes (scale bar: 500 nm); b: Nanoparticle tracking analysis (NTA) to quantify the size and concentration of exosomes (n = 3); c: Western blot analysis of protein CD63 enriched in exosomes.

We further isolated the exosomes from serum using the ultracentrifugation method, which is the first-line technique to isolate exosomes from the serum [33,34]. Due to the ultracentrifugation method can exist some extracellular vesicles, it is necessary to ensure the concentration and purity of the identified exosomes. Three methods (TEM, NTA, and WB) were used to characterize the isolated serum exosome. TEM was used to observe the structure and shape of the exosome. As shown in Figure 2a, the isolated exosomes were round and cup shape in media. NTA technology has been recognized as one of the characterizations of exosome. NTA technology was demonstrated that the size distribution of exosomes ranged from approximately 50 nm to 150 nm in diameter, and the average particle size is 127.8 ± 41.4 nm (Figure 2b). As shown in Figure 2c, CD63 is the specific exosome surface marker. Western bolt analysis was used to identify the exosome markers of CD63, which was enriched in the isolated exosome pellets at approximately 26 kD. All these results showed that exosomes had been purified adequately.

### 3.3 Fluctuation of HIF-1α expression in serum exosomes during the bacterial peritonitis

**Figure 3.**
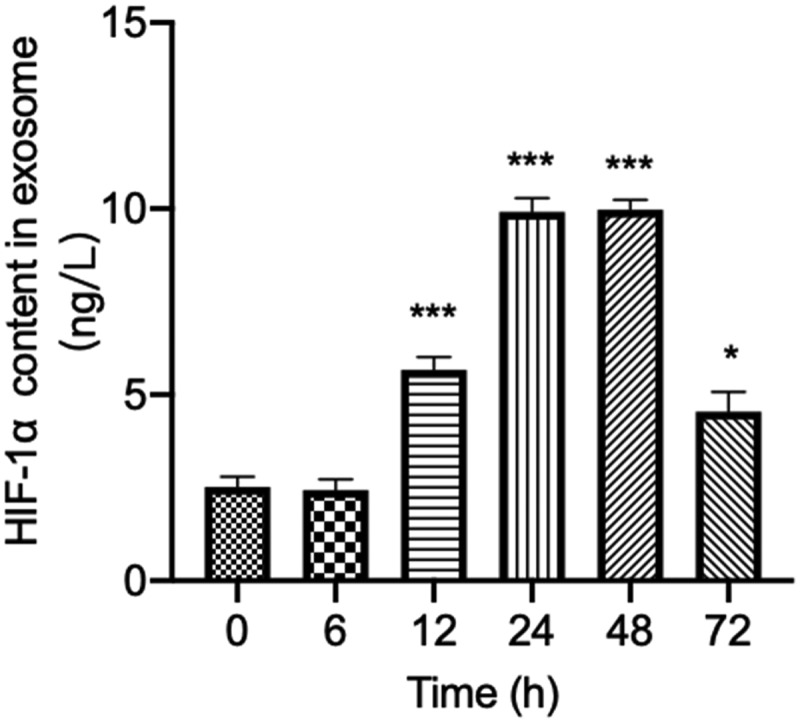
Changes of HIF-1α concentration in exosomes during 72 h after bacterial peritonitis induced by GFP-labeled *E.coli*. Experimental values are expressed as mean ± S.E.M *p < 0.05; **p < 0.01 and ***p < 0.001 compared with time 0 (n = 10 rats per group).

As the released HIF-1α in serum exosomes play an important role in tissue hypoxia after bacterial infection, the patterns of HIF-1α expression in serum exosomes through the course of the bacterial peritonitis episode within 72 h were investigated further. Expression of HIF-1α in serum exosomes of rats injected with GFP-labeled *E.coli* at 0 h as a baseline level. In line with the analysis shown in Figure 3, HIF-1α levels were enormously elevated in serum exosomes as early as 12 h after injection of GFP-labeled *E.coli*. It increased rapidly to reach the peaked level during 24–48 h of the episode. Subsequently, the expression of HIF-1α in serum exosomes returned to near baseline within 72 h. It suggested that the level of HIF-1α in serum exosomes might be the early potential biomarker in bacterial peritonitis. This result also indicated the importance of understanding the kinetics of HIF-1α expression in serum exosomes during bacterial peritonitis when planning sampling schedules. In summary, these findings suggested that levels of HIF-1α showed kinetics in serum exosomes in response to bacterial peritonitis and reiterate its potential as a promising biomarker in relation to early diagnosis of GFP-labeled *E.coli* induced bacterial peritonitis.

### 3.4 The external whole-body imaging of GFP-labeled E.coli and its response to ICS in bacterial peritonitis

**Figure 4.**
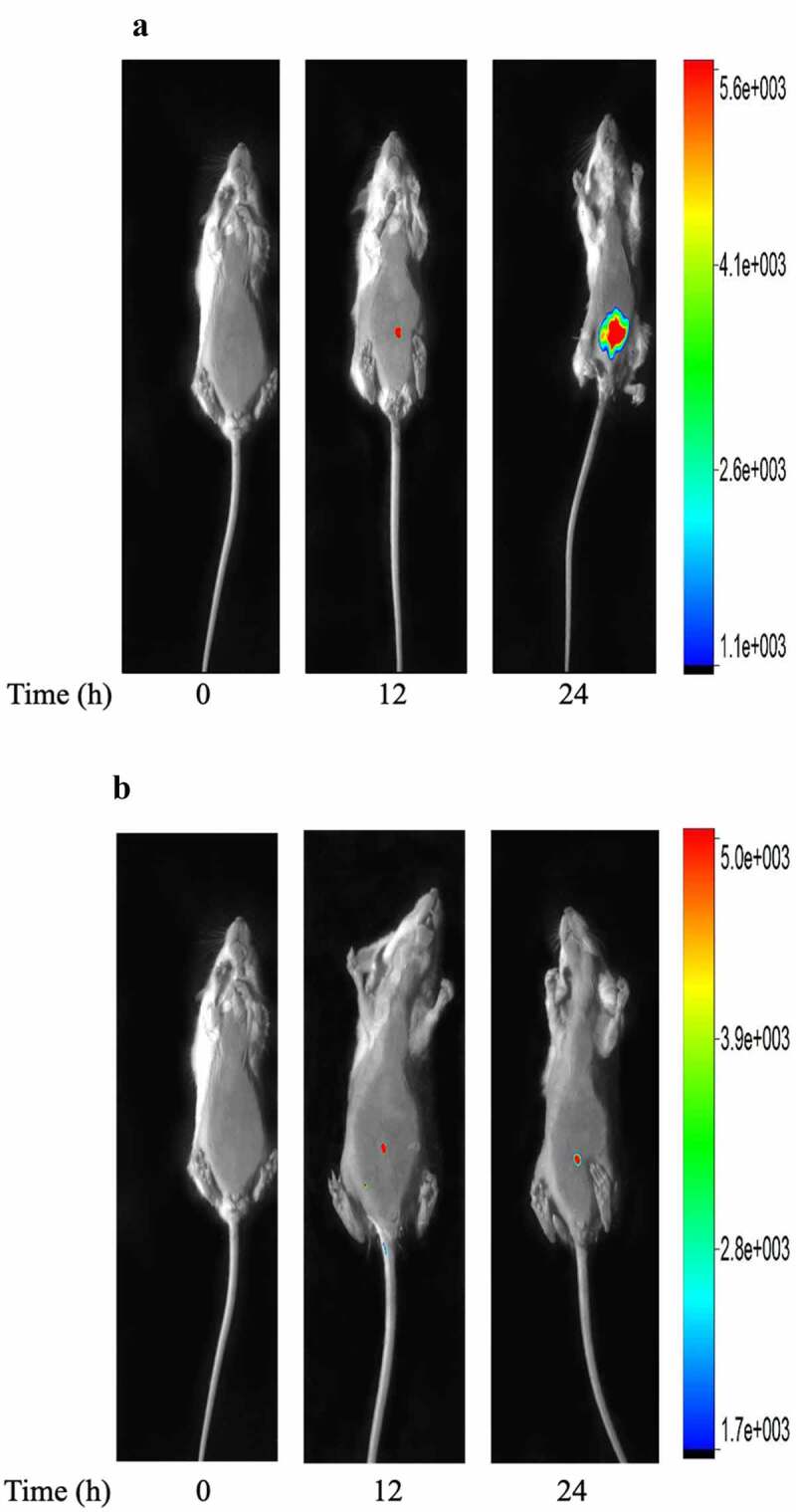
Whole-body imaging of GFP-labeled *E.coli* in bacterial peritonitis rat model. a: Representative images of injection 12 and 24 h after *E.coli*-GFP infection; b: Representative images of mice treated with ICS (20 mg/kg) at time points of 12 and 24 h after GFP-labeled *E.coli* infection. Animal survived. The fluorescence and bright field images are overlay. The right coordinate represents the fluorescence intensity.

ICS, as the broad-spectrum antibiotics, is used in clinical to treat various serious infections. To identify ICS treatment decreased bacteria’s growth, which might influence the tissue hypoxia environment with bacterial peritonitis. An authentic infection was established in mice by injection with GFP-labelled *E.coli*. The fluorescent of GFP-labelled *E.coli* was seen localized around the injection site by external whole-body imaging (Figure 4). 12 h later, the GFP-labelled *E.coli* was seen to spread throughout the peritoneum (Figure 4a). 24 h later, the degree of abdominal infection is significantly increased. The development of infection over 24 h and its regression after ICS treatment were visualized by whole-body imaging. ICS was demonstrated to reduce the degree of abdominal infection in time-dependent manner (Figure 4b). The results indicated that the effect of ICS reduced the growth of bacteria against bacterial infection since the treatment was shown to attenuate the degree of abdominal infection in the GFP-labelled *E.coli* induced bacterial peritonitis model.

### 3.5 Changes on the expression of HIF-1α in serum exosomes after ICS treatment in bacterial peritonitis model

**Figure 5.**
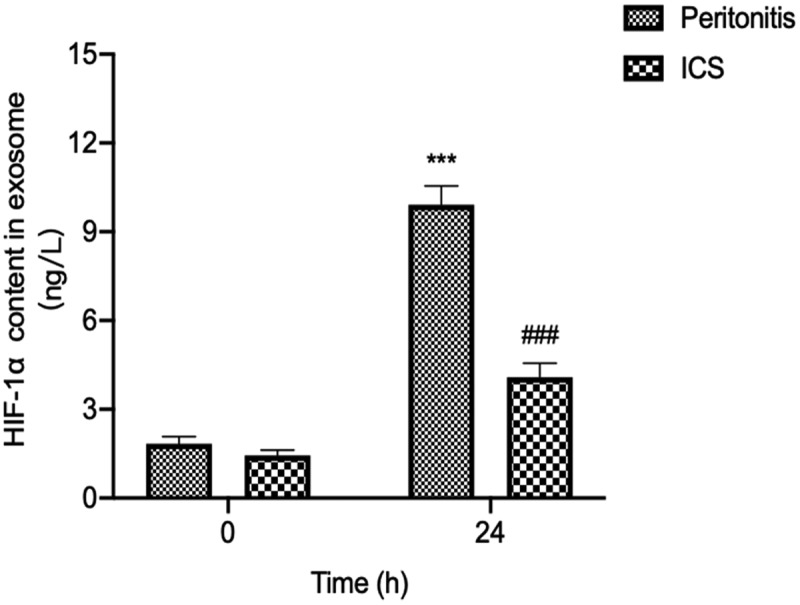
The effect of ICS on expression of serum exosomal HIF-1α in bacterial peritonitis model. Experimental values are expressed as mean ± S.E.M (n = 10 rats per group). **P < 0.01, and ***P < 0.001 compared with time 0. ^##^P < 0.01 and ^###^P < 0.001 versus model group at the corresponding time point (n = 10 rats per group).

To further analysis the ICS intervention might decrease the expression of HIF-1α in serum exosomes by reducing the growth of GFP-labeled *E.coli* in the abdominal cavity at 24 h after GFP-labeled *E.coli* injection. We detected the level of HIF-1α in serum exosomes at 24 h after ICS treatment. The results showed in Figure 5 that ICS was demonstrated to decrease the HIF-1α concentration in serum exosomes compared with the bacterial peritonitis model at 24 h (P < 0.001).

### 3.6 Decreased the serum concentrations of IL-1β and IL-6 after ICS treatment in bacterial peritonitis rat model

**Figure 6.**
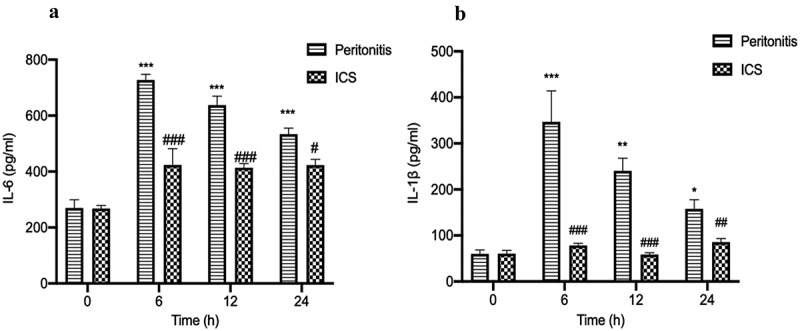
The effect of ICS treatment on the level of (IL-6 and IL-1β) in the serum with bacterial peritonitis model. a: The concentration of IL-6 in serum; b: The concentration of IL-1β in serum; Experimental values are expressed as mean ± S.E.M (n = 10 rats per group). *P < 0.05, **P < 0.01, and ***P < 0.001 compared with time 0. ^#^P < 0.05, ^##^P < 0.01, and ^###^P < 0.001 versus model group at the corresponding time point.

To further investigate the effect of ICS treatment on the expression of pro-inflammatory (IL-1β and IL-6) cytokines after GFP-labeled *E.coli* injection, we used ELISA kits to analyze the level of pro-inflammatory cytokines in the serum of bacterial peritonitis rat model during 24 h. The serum levels of pro-inflammatory cytokine (IL-1β and IL-6) were significantly increased in GFP-labeled *E.coli*-induced bacterial peritonitis rats during 24 h compared with the level of animals at 0 h (Figure 6). A significant decrease was detected in the serum level of IL-6 in *E.coli*-induced bacterial peritonitis rats treated with ICS for 24 h (Figure 6a). Meanwhile, the effect of ICS was statistically significant since the serum IL-1β level of ICS-treated groups during 6 to 24 h was recovered to the level of animals at 0 h (Figure 6b).

## 4. Discussion

Bacterial peritonitis is one of the leading causes of severe sepsis in the surgical intensive care unit [35]. It is reported in the literature that the most common pathogenic bacteria of peritonitis are Gram-negative bacteria, and most of the intestinal bacteria belong to this category [36]. The representative bacteria are *E. coli*. As the routine test for bacterial peritonitis (diagnostic paracentesis) is time-consuming and may involve serious abdominal wall hematoma, it is urgent to find an early diagnosis method for bacterial peritonitis through the noninvasive liquid biopsy. Serum exosomes play a future role in liquid biopsy. HIF-1α is a significant regulator of energy homeostasis in response to hypoxic and non-hypoxic stimuli, which has played an essential role in the development of infections by activating the host functional response, thus invading the pathogens [37]. Due to it being easily degraded under normal oxygen conditions, making it unstable in body fluids, thus leading to limitations of HIF-1α detection. The detection limit of existing methods (ELISA and WB) cannot reach the detection of HIF-1α in exosomes. Therefore, few studies on the expression of HIF-1α in serum exosomes at different time points of acute bacterial peritonitis. In this study, we first found that the dynamic changes in HIF-1α levels in serum exosomes during bacterial peritonitis via a new colorimetric method, the gold seed-coated with aptamer-functionalized Au @ Au core-shell peroxidase mimic, which was established by our team. ICS is a commonly used antibacterial drug in clinical. The pre-clinical application of HIF-1α as the potential biomarker was evaluated by analyzing rat serum exosomes from ICS intervention during bacterial peritonitis.

Bacterial peritonitis is the host’s systemic inflammatory response to bacteria [38]. During bacterial infection in the abdomen, it can activate the innate immune system and cause a series of inflammatory reactions. In rats with bacterial peritonitis, the pro-inflammatory factors (TNF-α, IL-1β, and IL-6) were increased due to the initiation of the inflammatory response. The high expression of pro-inflammatory factors (TNF-α, IL-1β, and IL-6) in the serum was closely related to bacterial peritonitis’s survival rate and progression [39,40]. The concentration of pro-inflammatory cytokines in serum directly reflects the severity of the host’s systemic inflammatory response [41]. The pro-inflammatory factors (IL-1β, IL-6, and TNF-α) concentrations in serum were significantly elevated at 6 h. Subsequently, the levels of these pro-inflammatory cytokines (IL-1β and IL-6) remained higher than the cytokine levels at 0 h. The dynamic changes of IL-6, TNF-α, and IL-1β levels are consistent with the excessive inflammatory response during bacterial peritonitis [40].

Hypoxia is the common microenvironmental feature in the inflammatory process associated with bacterial infections [42,43]. Increased oxygen demand and reduced oxygen supply are the main factors leading to tissue hypoxia during infection [44]. HIF-1α is the primary regulator of cell response to hypoxia. Many studies have shown that HIF-1α can also be increased in response to bacterial infections in addition to (HIF-independent) hypoxia-sensitive pathways [15]. HIF-1α is induced by bacterial infection and regulates the production of crucial immune effector molecules, including nitric oxide and TNF-α [43,45]. This study found that the concentration of HIF-1α in serum exosomes was significantly increased after 12 h GFP-labeled *E.coli* injection. The literature reported that mice deficient in HIF-1α in their myeloid cells reduced bactericidal activity and could not limit the systemic spread of infection [15,46]. Therefore, we suggested that the high expression of HIF-1α in GFP-labeled *E.coli*-induced bacterial peritonitis at this time point might be related to the bactericidal activity. Moreover, the high expression of HIF-1α can promote the release of inflammatory factors to increase the ability to kill the bacteria [47]. This result is consistent with the above-mentioned overexpression of pro-inflammatory factors detected in the serum at 12 h of GFP-labeled *E.coli* injection. The high expression of HIF-1α and inflammatory factors may be related to immune cells’ engulfing and killing bacteria. In the next 12 to 48 h, the expression of HIF-1α in serum exosomes has been continuously at high levels. The high expression of HIF-1α indicates that the host has enhanced the sensitivity to pathogens and the phagocytic activity of phagocytes to prevent more bacteria from entering the blood. Our finding estimated that the changes expression in serum exosomes of HIF-1α during the early acute phase of bacterial infection might be potentially consistent with the elevated pro-inflammatory cytokine markers during the process of bacterial peritonitis. Similarly, HIF-1α concentration in serum exosomes significantly decreased to baseline levels at 72 h. Meanwhile, the level of TNF-α at 48 h recovered to the level of cytokines at 0 h. HIF-1α might have important temporal roles during bacterial infection, regulating the inflammatory response during the early acute phase [19]. It might be used as a potential biomarker for diagnostic testing during the early acute phase of bacterial infection. This result also indicated the importance of understanding the kinetics of HIF-1α expression in serum exosomes during bacterial peritonitis when planning sampling schedules.

The potential pre-clinical application of HIF-1α as the biomarker was tested by analyzing rat serum exosomes from ICS intervention during bacterial peritonitis. We applied *In-Vivo Xtreme imaging system* to visualize the degree of abdominal infection. The results were showed that ICS treatment was shown to attenuate the degree of abdominal infection after GFP-labeled *E.coli* injection at 24 h. The present findings demonstrated that ICS possessed an antibacterial effect by attenuating the degree of abdominal infection. After ICS injection in bacterial peritonitis model, we also detected that ICS intervention reduced the expression of HIF-1α in serum exosomes at 24 h. ICS intervention might alleviate the degree of hypoxia microenvironment caused by bacterial proliferation through antibacterial activity. HIF-1α is a known transcription factor for various inflammatory cytokines, including IL-1β and IL-6 [48]. This study also confirmed that the level of pro-inflammatory cytokines (IL-1β and IL-6) significantly decreased in the serum of bacterial peritonitis rat model at 24 h. In particular, the effect of ICS intervention was found to be statistically significant since the serum IL-1β level of ICS-treated groups at 24 h was recovered to the level of animals at 0 h. In short, the quantitative expression and distribution of HIF-1α in serum exosomes during peritonitis might be used as an early biomarker to observe the process of bacterial peritonitis. It might provide a research basis for predicting and monitoring the hypoxic pathological process of acute bacterial peritonitis.

## 5. Conclusion

In this study, it is the first time to detect the dynamic changes in the quantitative expression of HIF-1α in serum exosomes as an early alternative biomarker of bacterial peritonitis. Subsequently, the pre-clinical application value of HIF-1α as the potential biomarker was evaluated by analyzing rat serum exosomes from ICS intervention during bacterial peritonitis. In short, all the results showed that HIF-1α concentration in serum exosomes of bacterial peritonitis might be provided basis research for further early predicting and monitoring the pathological process of bacterial peritonitis in pre-clinical.

## Data Availability

No data has been submitted to any open access databases. All data supporting the study is presented in the manuscript or available upon request.

## Highlights

1. A new colorimetric method applied in serum exosomal HIF-1α detection.
2. Pre-clinical evaluate diagnostic value of HIF-1α for peritonitis.
3. HIF-1α decreased with infection reduction for the antibiotic therapy.

## Abbreviations

**HIF-1α**: Hypoxia-inducible factor-1 alpha
**ICS**: Imipenem and Cilastatin Sodium
*E.coli*: Escherichia coli
**CIAOW**: Complicated Intra-Abdominal Infection Observational Worldwide
**SPF**: Specific pathogen-free
**GFP**: Green fluorescent protein
**LB**: Luria–Bertani
**CFU**: Colony-forming units
**IL-6**: Interleukin 6
**IL-1β**: Interleukin 1beta
**TNF-α**: Tumor necrosis factor-alpha
**WB**: Western blot
**ELISA**: Enzyme-linked immunoassay
**PBS**: Phosphate buffered saline
**TEM**: Transmission Electron Microscopy
**NTA**: Nanoparticle Tracking Analysis
